# Development and Validation of the Amharic Version of Self-Efficacy and Outcome Expectancy Measures on Intention to Take Preventive Actions on Noncommunicable Disease

**DOI:** 10.1155/2023/6669157

**Published:** 2023-10-31

**Authors:** Shumye Molla Legesse, Habtamu Wondimu

**Affiliations:** ^1^Department of Psychology, College of Social Sciences and Humanities, Debre Birhan University, Ethiopia; ^2^School of Psychology, College of Education and Behavioral Studies, Addis Ababa University, Ethiopia

## Abstract

This study is aimed at developing and accessing the validity and reliability of an Amharic version of the self-efficacy and outcome expectancy measures on noncommunicable disease prevention strategies. The intentions to take protective measures on NCDs' self-efficacy and outcome expectancy scales were created in Amharic using a sequential nine-step process that included translation and contextualization of the items, content validity, pretesting of the questions, sampling, and survey administration. Principal component analysis was conducted on 829 university students which showed a one-factor solution for self-efficacy and a three-factor solution for outcome expectancy scales using split-half measures. Confirmatory factor analyses supported the factor structure, which also demonstrated good internal consistency (.828 self-efficacy, .766 outcome expectancy). The scales had a moderate level of correlation (*r* = .35, *p*.001) between them. The study resulted in reliable and valid Amharic versions of self-efficacy (9-item) and outcome expectancy (12-item) scales.

## 1. Introduction

Noncommunicable diseases (NCDs) contribute to 41 million deaths annually, which is equivalent to 74% of all deaths globally [1]. Each year, 17 million people die from a NCD before age 70, out of which 86% occur in low- and middle-income countries. Of all NCD-related deaths, 77% are in low- and middle-income countries [2]. Cardiovascular diseases account for most NCD-related deaths (17.9 million people annually), followed by cancer (9.3 million), chronic respiratory diseases (4.1 million), and diabetes (2.0 million including kidney disease deaths caused by diabetes). These four groups of diseases account for over 80% of all premature NCD death [3]. Tobacco use, physical inactivity, the harmful use of alcohol, and unhealthy diets increase the risk of dying from NCD [4]. Following positive lifestyle, detection, screening, and treatment of NCDs, as well as palliative care, are key components of the response to NCDs. Similarly, Ethiopia encountered a sizable burden and an immoderate capacity to grow exposure to important NCDs' hazard elements within the future (“Are the researchers ready for the rising silent epidemic of metabolic syndrome and chronic noncommunicable disease in Ethiopia?”) [5].

NCDs accounted for more than 52% of deaths in Ethiopia [6–8]. The risks of death from one of the NCDs (cardiovascular illnesses (CVDs), diabetes, maximum cancers, and chronic respiratory illnesses) have become about 24% in people between 30 and 70 years of age [8]. The national and international literature additionally highlighted an increase and diversification in the NCDs' risk factors, together with high blood pressure and obesity/weight issues (Desta, Seyoum, and Sharew, “Emerging public health problems in Ethiopia: chronic non-communicable diseases”) [9]. The Ethiopian STEP survey conducted in 2014 found that 12% of the people between the ages of 40 and 64 years had already had one form of CVD or an immoderate diagnosis (i.e., ≥30%) of 10-365-day CVD danger [7]. Those findings support the countrywide issue of growing risk factors for NCDs.

Self-efficacy has generally been proven to predict intentions and behaviors in several health-functioning domains [10]. Positive correlations exist between the intention to engage in a particular beneficial behavior and the behavior itself [11]. Efficacy beliefs influence how one evaluates their resources in difficult situations and help them establish behavioral intentions [12]. The more firmly people are committed to engaging in the desired activity, even in the face of failures, the higher the objectives they set for themselves and the better they engage in healthy behavior [13–16].

Furthermore, according to Ayele [17], one of the major factors contributing to the behaviors identified in Bandura's social cognitive theory is result expectancy. Expectations regarding negative effects may have a significant role in determining drinking behavior in addition to positive expectancies about the intended effects of alcohol, which are the focus of alcohol expectancy research [18–23].

Positive expectancies also refer to reasons for drinking, while negative expectancies pertain to reasons for avoiding drinking or for not drinking as much [24]. According to Becker and Joseph [25], drinking was associated with both positive and negative expectations about its effects (such as negative emotional alterations and loss of control) ([26, 27]; and [28]). On the other hand, Stewart et al. [29] discovered that intentions and behavior were connected to positive expectancies, but not negative ones.

### 1.1. Theoretical Foundations

Theory-based interventions are successful in altering a wide range of health behaviors, including smoking, physical exercise, and organ donation [30]. Increased access to green space, prescriptions from actors, and programmatic activities have been the main treatments to date to enhance time spent in nature [31, 32]. A crucial initial step in creating theory-based interventions is the creation of valid and trustworthy assessments of theoretical notions [33]. These and other researches highlight the necessity of creating psychosocial measures that are accurate and reliable to assist theory-based interventions to improve involvement in preventive behavior [34].

Two of the strongest theoretical constructs for predicting behavioral intentions are self-efficacy and outcome expectancy. The theories of planned behavior, health belief, and social cognitive theory all incorporate self-efficacy [33, 35, 36].

The main concept in both the theory of planned behavior and the theory of reasoned action is intention. Intentions are the primary construct via which attitudes, norms, and self-efficacy influence behaviors, according to Fishbein [35]. Perceived competence to carry out a behavior successfully is a component of self-efficacy [37]. Self-efficacy, which is influenced by personal qualities and environmental circumstances, encompasses both the capacity to carry out an action and the ability to control hurdles [38]. Several pieces of research [39, 40] have demonstrated that self-efficacy is among the best predictors of intention. For instance, Netz and Shulamith [40] discovered positive correlations between high self-efficacy levels and engaging in physical activity, indicating that perceived self-efficacy in one's capacity to engage in physical activity must be established before other motivational interventions are taken into consideration [41].

Therefore, it is likely that self-efficacy is required to raise involvement in preventive activities; nevertheless, measures are required to confirm this link. The closest indicator of a healthy activity is the intention to engage in it [42]. It has been demonstrated that attitudes, norms, and self-efficacy all affect intentions and, through modifications in intentions, behavior [39–43]. According to the theory of planned behavior, intentions, which act as a mediator for all other routes, directly affect behavior [42]. The measurement of preventive behavior intentions is crucial because they should be directly influenced by changes in self-efficacy, attitudes, and norms [44].

The study's outcomes contribute to the broader field of noncommunicable disease prevention and health promotion by providing valuable insights and evidence on effective strategies, interventions, or approaches that can be implemented to address these diseases. The findings of the study may help inform public health policies, guidelines, and programs aimed at preventing noncommunicable diseases and promoting overall health.

By understanding the specific factors or behaviors that contribute to noncommunicable diseases, such as unhealthy diet, physical inactivity, tobacco use, or excessive alcohol consumption, the study's outcomes can guide efforts to develop targeted interventions. For example, if the study identifies a particular dietary pattern associated with an increased risk of a specific noncommunicable disease, this information can be used to develop educational campaigns promoting healthier eating habits.

Additionally, the study's outcomes may also highlight gaps in knowledge or areas for further research within the field of noncommunicable disease prevention and health promotion. This can help guide future studies and investigations aimed at better understanding the underlying causes of these diseases and developing more effective prevention strategies.

Overall, by contributing new knowledge and evidence-based recommendations, the study's outcomes play a crucial role in advancing our understanding of noncommunicable diseases and informing efforts to prevent them and promote better health outcomes for individuals and communities.

### 1.2. Study Objectives

The purpose of this study was to provide a valid and accurate Amharic version of self-efficacy and outcome expectation scales for intentions to take preventive actions against NCDs.

## 2. Methods

### 2.1. Design

The sequential procedures created by Jackson [43] and Comrey [44] and improved upon by Boateng et al. [45] were used to produce the IPMNCD self-efficacy and outcome expectancy scales. The nine steps of these methods are as follows: (1) domain identification and item generation, (2) content validity analysis, (3) pretesting of questions, (4) sampling and survey administration, (5) item reduction, (6) factor extraction, (7) dimensionality tests, (8) reliability tests, and (9) validity tests.

Self-efficacy was described in phase one as a person's belief in his or her capacity to act and to continue acting in the face of difficulties or challenges related to engaging in preventative behaviors [46]. Expected results or repercussions of engaging in preventive behavior on the probability of acquiring NCDs were described as outcome expectancy (Maddux, Norton, & Stoltenberg,1986).

The effects of these expectations on a variety of health behaviors, such as alcohol intake, smoking, and weight management, have been thoroughly investigated in behavioral medicine [47]. Plans to engage in specific preventive activities in the near future were used to describe intentions [48]. Based on these cues, the researchers independently created measures regarding self-efficacy and outcome expectancy to spend time engaging in preventative behaviors. The principal investigator, who removed duplicates aside from those utilized as validity checks, examined all generated items.

During phase two, an expert team survey was used to review and rate each item. On a three-point Likert scale, from not relevant to extremely relevant, items were first evaluated for their relevance to the construct of self-efficacy or result expectancy. Then, thematic subgroups were ranked according to how crucial they were for practicing preventive behaviors [49]. Items that scored in the bottom quartile of importance and had relevance means less than 2.5 were eliminated.

In phase three, the researchers enlisted 100 Debre Birhan University graduates to take part in a pretesting exercise using a cognitive interview. For questions that were unclear, double-barreled, or otherwise challenging to answer, the principal researcher sat next to the respondent and collected feedback item by item [32]. During this round of the trial, items that performed poorly were once more eliminated.

Phase four involved the acquisition of a sample of student volunteers using a disproportionate stratified random sampling. The number of respondents required for factor analysis varies, ranging from 5 to 10 for each item to 100 to 1000 for each research. A sample size of 1000 has been evaluated as excellent for factor analysis stability by Comrey [44].

The sample size for this study was 1000 because split-half procedures were being used. To be included in the survey, respondents were enrolled in any of the standard degree programs. A more random sample approach was used to reduce selection bias. To be representative of those traits, respondents were stratified by age, gender, study year, and location of birth (urban vs. rural) over two weeks (November 23–December 6, 2022). Before doing the survey, participants were given a consent information page and asked to verbally confirm their consent.

The survey typically took 35 minutes to complete. The World Medical Association Declaration of Helsinki and the ICMJE Recommendations for the Conduct, Reporting, Editing, and Publication of Scholarly Work in scholarly journals were both followed in all study processes. The Addis Ababa University Institutional Review Board approved the research proposal and all the procedures for the study.

### 2.2. Materials/Measures

Demographic information and social cognition assessments are included in the instrument's contents. When it was practical, questions were formatted using the same framework and were adopted from trusted instruments.

### 2.3. Sociocognitive Measures

All of the self-efficacy questions began with the phrase “I am confident that I can take steps to prevent NCDs.” There were five possible answers which included strongly disagree, disagree, neither agree nor disagree, agree, and strongly agree. Items with outcome expectations began with the stem: “.......... Highly disagree, disagree, neither agree nor disagree, agree and strongly agree were all acceptable responses to the question ‘Prevents NCDs'.”

### 2.4. Sociodemographic Data

Age, gender, year of study, and location of birth (rural vs. urban) were among the sociodemographic factors assessed in this study.

### 2.5. Participants

1000 participants consented to participate in the survey phase of the study. Informed consent was given by 850 of them, and 829 of them responded to all inquiries. Eighty-two percent of those who started the survey, or 829 people, finished it and passed the quality check. As indicated in Table 1, participants self-identified as male are 59.6% and as female are 40.4%. Just over half of the sample (53.1%) were born in cities, with a median age of 22 years (SD = 1.54). Participants' study years were distributed as 51.4% in their second year, 24.8% in their fourth year, 13.5% in their third year, and 10.3% in their fifth year of study.

### 2.6. Data Analysis

In the study's fifth phase, we carried out item analysis. Items in surveys correlate with one another at a moderate level (>.70). Collinearity was therefore not a problem. All items passed the recommended level of scrutiny for extreme distribution characteristics like a noncentral mean, restriction in range, skewness, and kurtosis.

The factor dimensionality of the scales was investigated in phase six. We applied the split-half method, in which the sample was randomly split in half, to enable both exploratory and confirmatory analyses. For exploratory analysis, the sample's first half was chosen. Using pair-wise deletion, a matrix of item intercorrelations was created from the first half of the sample, and this matrix was then subjected to an exploratory principal component analysis (PCA).

By contrasting the outcomes of two procedures—the screening process and the parallel analysis method—that have been proven to be reliable predictors of the right dimensionality of an item set, the number of components to keep was determined [50].

Because the screening approach of Horn [51] may sometimes excessively extract factors, parallel analysis tables by Lautenschlager [50] that also employed orthogonal rotational (varimax) were looked at. Items that are loaded on a factor of less than .50 were eliminated [52]. To lessen colinearity between the subscales, items loading greater than .30 on several components were also eliminated.

Confirmatory factor analysis (CFA) was carried out on the second half of the data in phase seven. This was done after PCA was conducted on the first half of the data to assess the latent structure. CFA was conducted to give evidence for construct validity by evaluating how accurately the variables or items of the measures represent the constructs; CFA offers a rigorous test of the suggested scales [53]. Amos 24 was used to specify indicators and estimate parameters using the maximum likelihood method.

An analysis of the entire data fit is necessary for CFA evaluation [54]. In step seven, CFA was used to demonstrate convergent validity by comparing the variance of the concept's items to that of the latent construct. To demonstrate convergent validity, Hair et al. [55] advise that all factor loadings be statistically significant with loadings of at least .50 or higher. When the quantity of information exchanged with a latent construct is more than the error variance, Fornell and Larcker's [53] state parameter estimates of .70 or higher are acceptable. Additionally, the CFA permits the assessment of extracted average variance (AVE). According to Hair et al. [55], AVE ought to be greater than .50. The third convergent validity criterion is reliability, which is measured by Joerskog's rho. Values higher than .7 imply internal consistency, which means that all of the scale's questions consistently measure the same latent concept [54].

In phase eight, Cronbach's alpha was used to assess the reliability of the scales. A good indicator of internal consistency is generally thought to be an alpha > .70 [56]. Individual items were assessed to determine whether eliminating the item would raise the scale's overall alpha if the overall alpha fell below .70. The Pearson correlation coefficient was used in phase nine to analyze the correlations between self-efficacy, outcome expectancy, and intentions and to assess criterion validity.

### 2.7. Ethical Review

The proposal for this paper was presented at Addis Ababa University, College of Education and Behavioral Studies, School of Psychology. Approval was received from the school head by letter number 5/150/2013. The data were analyzed anonymously. Permission was obtained from the administration of Debre Birhan University which was the data collection site. Before interviews were conducted, all interviewees were provided with adequate information about the purpose of the study, the contents of the interviews, and the contact details of the principal investigator (PI). Verbal consent was obtained from each informant before they were interviewed.

## 3. Results

The research team developed a total of 25 original self-efficacy items and 33 original outcome expectancy items during phase one. In phase two, the self-efficacy item set was reduced to 11 items, while the outcome expectancy item set was reduced to 16 items. During phase three, cognitive interviews were conducted with a diverse group of 100 individuals, varying in age, gender, and location, to pretest the items. Further refinement occurred during pretesting, resulting in the reduction of the self-efficacy set to 10 items and the outcome expectancy set to 12 items.

The quality of the items was assessed after completing the entire survey. Nine self-efficacy measures with good variance were retained, as correlations between each item and other items were all below .70. The intention to take preventative measures was then evaluated for specific objects. Only one item was removed due to weak connections with the desire to take precautions, leaving nine items in place. Conversely, all 12 result expectancy items exhibited good variance and were retained.

The principal component analysis (PCA) was conducted on the initial 420 participants (*n* = 420) in phase six of the study. In the self-efficacy domain, two components had eigenvalues greater than one (3.824, 1.255), indicating the presence of two factors according to both the scree approach by Horn [51] and Lautenschlager's tables [50]. Both options were explored, but some items were loaded on both factors in the two-factor solution, making it difficult to interpret. In the one-factor solution, items with loading greater than .50 accounted for 42.492% of the variation.

In the outcome expectancy model, three components (3.501, 1.986, and 1.307) had eigenvalues larger than one, explaining a total of 56.659% of the variation. Questions 1, 2, 3, 4, 5, and 9 were loaded onto factor one in the rotational component analysis. Conversely, questions 10, 11, and 12 were loaded onto factor three, while questions 6, 7, and 8 were loaded onto factor two. Each item contributed significantly to its respective component with loadings exceeding .50. Factor one primarily focused on general health outcomes as all its loading factors were related to this domain. Factor two represented behavioral outcomes as all its loading factors were associated with behavior-related aspects. Factor three captured cognitive dilemmas as all its loading factors pertained to cognitive challenges.

Consequently, component one can be described as a motivator for overall health improvement, while factor two serves as a motivator for behavioral changes. Factor three acts as a cognitive barrier that hinders progress toward desired outcomes.

**Figure 1:**
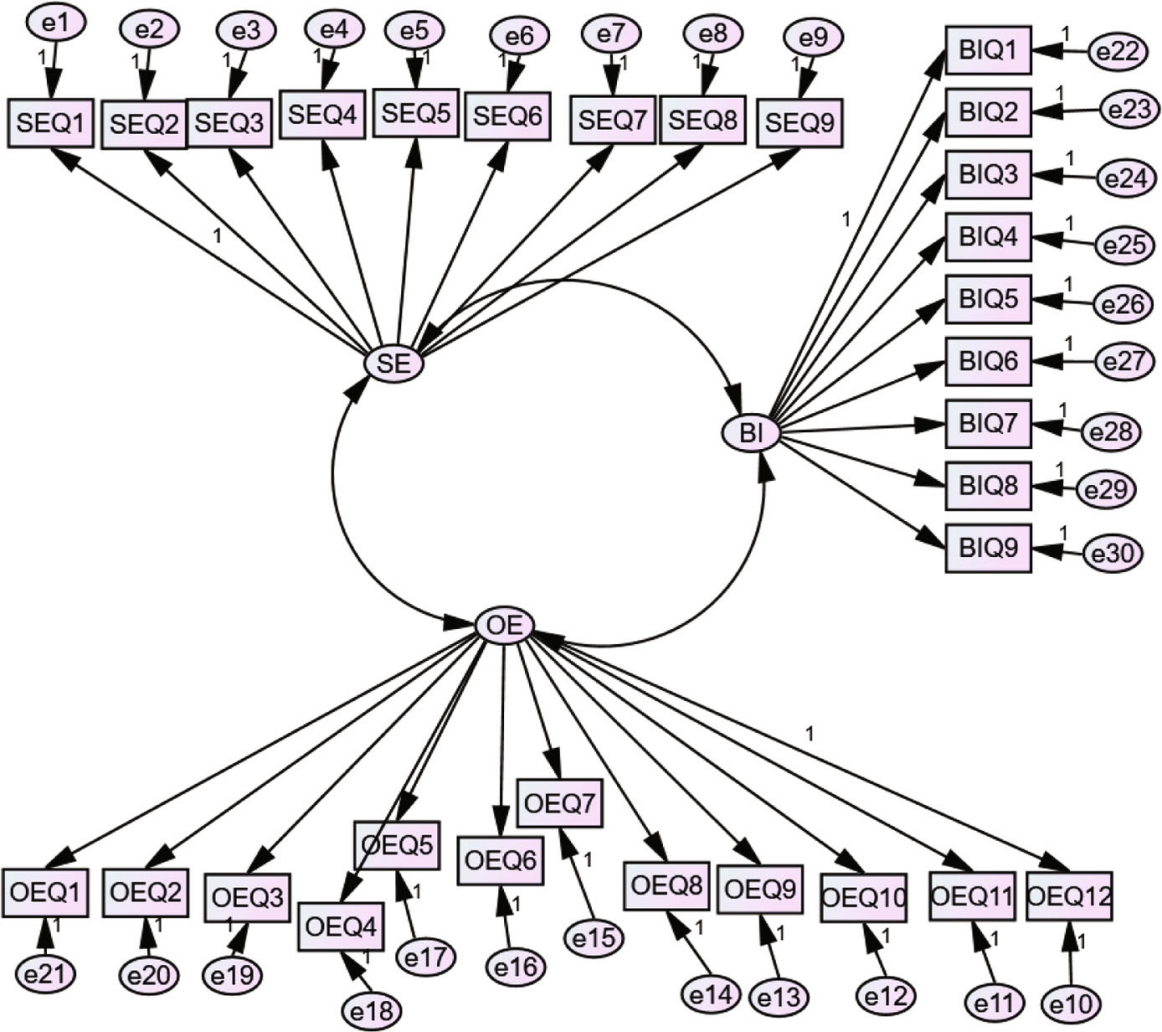
The measurement model of the variables SE, OE, and BI. SE = self-efficacy; OE = outcome expectancy; BI = behavioral intention; Q = questions/items; e = error terms. Note: the decimal numbers on each line represent the standardized factor loadings.

Convergent validity was established through confirmatory factor analysis (CFA) conducted in phase seven. The standardized loadings for items assessing self-efficacy ranged from .573 to .752, all of which were statistically significant at *p* < .001. Furthermore, the CFA revealed that the average variance extracted (AVE) from these items was .695, surpassing the recommended cutoff value proposed by Hair et al. [55]. This finding further supports the convergent validity of the measure. The CFA model for the study variables is illustrated in Figure 1.

Reliability, another criterion for convergent validity, was assessed using the Joreskog rho construct and Cronbach's alpha coefficient [54], which yielded a value of .894. A coefficient above .7 indicates that all components of the scale consistently measure the same underlying concept [54].

After conducting the CFA, fit statistics were evaluated to assess how well the model fit the data. The results indicated an adequate fit with comparative fit index (CFI) = .936, Tucker‐Lewis index (TLI) = .914, standardized root mean square residual (SRMR) = .046, and root mean square error of approximation (RMSEA) = .087.  Table 2 presents these findings. For items assessing outcome expectancy, significant standardized loadings ranging from .255 to .848 were observed at *p* < .001. Convergent validity was demonstrated by an AVE of .567 and construct dependability by a rho coefficient of .882. However, it is worth noting that the model only provided a fair fit to the data.

Cronbach's alpha coefficients were calculated to assess the internal consistency of the self-efficacy and outcome expectancy scales in phase eight, yielding values of .865 and .776, respectively. Furthermore, a correlation analysis revealed a significant positive correlation of .350 (*p* < .001) between the two scales. In phase nine, no significant differences were observed on either scale when considering variables such as gender, place of birth, or academic field of study. These findings are summarized in Table 3.

To evaluate the convergent and divergent validity of the scales with demographic variables, age, sex, and academic field, additional analyses were conducted. The results indicated a weak positive correlation between self-efficacy and outcome expectancy (*r* = .350, *p* < .001). Self-efficacy was not found to be associated with age (*r* = −.062, *p* > .05), study year (*r* = .003, *p* > .05), or intention (*r* = .559, *p* = .001). Similarly, outcome expectancy was correlated with intention (*r* = .281, *p* = .001), but not with age (*r* = −.074, *p* > .05) or study year (*r* = −.021, *p* > .05). Notably, age and study year exhibited a moderate level of correlation (*r* = .471, *p* = .001).

Regarding gender and place of birth as factors influencing self-efficacy and outcome expectancy scores, no significant differences were found. Additionally, there were no discernible differences in self-efficacy scores among different college student groups. However, a significant difference was observed in the types of college students who participated (*F*(1) = 8.358, *p* = .004), with an effect size of *η*^2^ = .020.

Furthermore, when examining self-efficacy scores based on study year and level of intention, significant differences were found (*F*(3) = 3.238, *p* = .022; *F*(1) = 76.423, *p* < .001), with effect sizes of *η*^2^ = .023 and *η*^2^ = .158, respectively. Similarly, outcome expectancy scores demonstrated significant differences based on level of intention (*F*(1) = 32.899, *p* < .001) and study year (*F*(3) = 2.684, *p* = .046), with effect sizes of *η*^2^ = .019 and *η*^2^ = .075, respectively. For a comprehensive overview of the results, please refer to Table 4.

## 4. Discussion

The purpose of this study was to develop reliable and valid Amharic versions of questionnaires measuring self-efficacy and outcome expectations for intentions to prevent NCDs. It will help with health promotion and assessment efforts to have a better knowledge of the cognitive variables that influence people to take protective measures against NCDs. The investigation adhered to the gold standard recommendations put forth by Boating et al. [45]. In confirmatory factor analysis, a one-factor solution for self-efficacy and a three-component solution for result expectancy were found for the scales. Internal consistency was quite high for both measures. There was a fair amount of correlation between the scales.

These findings have substantial implications for evaluating the relationship between social cognitive factors and health, including new understandings of how perceived affective reactions to take preventive measures against NCDs and influence propensity for frequency and duration of health activity. Overall, both self-efficacy and outcome expectations were favorably correlated with intentions to adopt preventative actions against NCDs.

A focus on measuring and boosting confidence to take protective measures in a variety of situations, as well as addressing expectations when engaging in protective measures, is one of the recommendations for developing interventions to increase the likelihood of taking protective measures. As a result, depending on the particulars that make up an intervention's focus, strategies will take a range of distinct forms. A comprehensive review and meta-analysis indicated that action planning, time management, rapid self-monitoring of behavioral outcomes, and preparing social support and societal change were all effective ways to boost self-efficacy for physical exercise [57]. Increases in connectedness to efforts or the meaning associated with them can be made through guided experiences that increase self-efficacy in taking protective measures, for instance, while effective increases in results and expectations can be made through goal-setting, planning, removing obstacles, and raising awareness [58].

The scales and demographics revealed a number of intriguing conclusions. Good intentions have been demonstrated to support healthy aging, while age and both self-efficacy and result expectancy were adversely associated [59]. Mobility or safety issues may diminish older individuals' goals and self-efficacy to spend more time exercising. Less than 10% of park visitors in the US are senior citizens, who may benefit from parks designed with them in mind [60, 61]. Similar results were observed for general health, with healthier individuals expressing stronger self-efficacy and intentions.

Although there was no difference in intentions by gender, men reported having stronger self-efficacy. Females enjoyed outside areas and reported being more connected to healthy activities, but they were less likely to engage in nature-based recreation, according to a recent study [49]. There may be some variations in self-efficacy behind this. Racial and ethnic differences were minimal; there were no variations in self-efficacy, and the only group with higher result expectancies was young adults who were born in cities.

Both self-efficacy and outcome expectancy were found to be significantly impacted by the degree of intentions toward taking preventative measures. This offers a preliminary understanding of the need of increasing good intentions while offering solutions to worries about protective measures.

Interventions that promote wholesome or pleasant lifestyles have gained popularity during the past few years. However, they have relied on introducing vegetation to indoor and urban spaces, doctor referrals (such as Entoto Park), or location-based programming (such as community gardens) [62–65]. In our country, Ethiopia, there are not many theory-based behavioral change therapies that focus on people, families, or other social groups.

The supply of measurements that will serve as the basis for treatments about self-efficacy and outcome expectancies to implement preventive measures is advanced by this study. The subsequent elimination of items based on factor loadings resulted in the creation of brief, approachable measurements that may be used in a range of academic and real-world settings.

There are a few limitations to this study. Although the sample included students from all colleges at Debre Birhan University and was representative of the student population in terms of gender and academic year, the respondents' average age tended to be lower than the median for the population. Students that were not on their respective campuses during the data collection were not included in this study. The self-efficacy scale does deviate slightly from Bandura's [60] suggestions, which called for adopting a 10-point Likert scale and responding with “certainty” rather than “confidence” in place of “assurance.” However, the majority of self-efficacy assessments in the literature on health promotion use a 5-point Likert scale similar to this study [66, 67]. Moreover, the authors collected one-time point data from 829 student participants and split the data to conduct both exploratory and confirmatory factor analysis. Using the same sample for both exploratory factor analysis (EFA) and confirmatory factor analysis (CFA) is generally not recommended. If the same sample is analyzed using two different methodological approaches and produces conflicting results, it suggests that the issue lies in the methodologies themselves rather than the sample data. To address this, researchers can consider collecting data at different times and locations or through different mediums to obtain two distinct samples. However, this approach may introduce biases and make the samples incomparable. For instance, a sample collected from Facebook and another from TikTok are likely to represent different populations. To mitigate this, researchers should aim to identify multiple sources for obtaining samples and ensure that each sample includes participants from all identified sources. By combining all participants from different sources into a single large sample and then splitting it into two subsamples using an appropriate method, both subsamples would contain participants from all sources [68] provide an example of this approach).

In conclusion, this study has yielded valid and reliable measures of self-efficacy and outcome expectancy to take protective measures from NCDs in the Amharic language. The measurements will be valuable in creating and assessing theory-based treatments to boost protective behaviors in the Ethiopian context.

## Figures and Tables

**Table 1:** Sample demographics.

Variable	Sample M (SD) or %
Gender (male, %)	40.4
Age (*M* and SD)	22 (1.54 SD)
College	
Health (%)	39
None health (%)	61
Birthplace	
Urban (%)	53.1
Rural (%)	46.9
Study year	
2^nd^ year (%)	51.4
3^rd^ year (%)	13.5
4^th^ year (%)	24.8
5^th^ year (%)	10.3

**Table 2:** Confirmatory factor analysis results.

Constructs and measurement item	S.L.	S.E.	Rho
Self-efficacy (*α* = .865; *ρ* = .894; AVE = .695)			
I am sure how to prevent noncommunicable diseases	.670	.046	.449
I can follow a healthy lifestyle to prevent noncommunicable diseases	.770	.030	.593
I can monitor health to prevent noncommunicable diseases	.710	.037	.548
I have information on how to prevent noncommunicable diseases	.580	.053	.336
There are many things I can do to reduce my risk of contracting NCDs	.690	.037	.476
I am sure how to prevent noncommunicable diseases	.660	.051	.436
I can actively exercise to prevent noncommunicable diseases	.620	.051	.384
I can implement a healthy diet to prevent noncommunicable diseases	.590	.044	.348
I can use drugs to prevent noncommunicable diseases	.560	.049	.314
Outcome expectancy (*α* = .776; *ρ* = .882; AVE = .567)			
I often try to take preventive measures because I am always ill	.720	.052	.518
Getting infected with NCDs can lead to dangerous consequences	.710	.057	.548
Preventive measures help prevent noncommunicable diseases	.540	.074	.292
Taking preventive measures will save us from regret later	.620	.077	.384
It is important to take preventive measures to avoid contracting NCDs	.850	.076	.723
Following a healthy diet prevents noncommunicable diseases	.710	.082	.548
Losing weight can prevent noncommunicable diseases	.490	.096	.240
Not smoking reduces the risk of contracting noncommunicable diseases	.690	.079	.476
Regular health check-ups are useful to prevent noncommunicable diseases	.640	.048	.410
I doubt it would be useful to take any preventive measures	.760	.048	.578
It takes so much effort that I do not feel compelled to take any preventative measures	.260	.050	.068
I do not believe that any medicine will not be effective in preventing NCDs	.580	.057	.336

Entries are standardized values; all statistically significant (*p* < .01). Error variance entries are standardized. *α* = Cronbach's alpha of reliability; *ρ* = composite construct reliability; AVE = amount of variance extracted; Rho = item level reliabilities; S.L. = standardized factor loadings.

**Table 3:** Pearson's product moment correlation.

	Self-efficacy	Behavioral intentions	Outcome expectancy	Age
Behavioral intentions	.559^∗∗^			
Outcome expectancy	.349^∗∗^	.281^∗∗^		
Age	-.062	.025	-.074	
Study year of students	.003	.049	-.021	.471^∗∗^

^∗∗^Significant at *p* = 0.00; - = negative or opposite relationship.

**Table 4:** *T*-test of self-efficacy and outcome expectancy by intention to take protective measure, sex, college, birthplace, and study year.

	Sum of squares	df	Mean square	*F*	Sig.
Self-efficacy by sex, birthplace, colleges joined, study year, and level of intention					
Birthplace	61.771	1	61.771	1.628	.203
Sex	52.741	1	52.741	1.389	.239
College	63.277	1	63.277	1.668	.197
Study year of students	363.121	3	121.040	3.238	.022
Level of intention	2450.600	1	2450.600	76.423	.000
Outcome expectancy by sex, birthplace, colleges joined, study year, and level of intention					
Sex	105.673	1	105.673	2.080	.150
Birthplace	68.613	1	68.613	1.348	.246
College	418.210	1	418.210	8.358	.004
Study year of	405.199	3	135.066	2.684	.046
Level of intention	1554.382	1	1554.382	32.899	.000

Sig. = significance level; df = degree of freedom.

## Data Availability

All relevant data are within the paper, and its supporting information files are available on reasonable request to the corresponding author.
